# Treatment of Cancer (4th edn)

**DOI:** 10.1038/sj.bjc.6600964

**Published:** 2003-05-13

**Authors:** Vanessa Gill

**Affiliations:** 1 St James's University Hospital, Leeds, UK

Covering the entirety of the treatment of cancer in one text would seem a rather ambitious undertaking. The fourth edition of this book manages to cover the basics of the subject, while including updates on the newer developments in cancer treatment.

While the content is mainly of relevance to the clinical oncologist, there is much that would appeal to doctors and the allied professions working in other specialities, including medical oncology, palliative care medicine as well as general medical and surgical fields.

The book is divided into three sections. The first section is entitled Principles. Molecular biology and radiobiology are covered in detail, with reference to standard texts in use by the clinical oncologist preparing for the part 1 FRCR (e.g. Steel, Basic clinical radiobiology). There is a comprehensive overview of the basic sciences including clinically relevant information and results of recent trials. Radiotherapy trials on altered fractionation are discussed, as is the latest thinking on the relevance of tumour hypoxia to radiation response. The radiobiological models in use are clearly explained, including the concept of biologically effective dose (BED), along with the equations used and some worked examples.

There is a chapter on the use of hyperthermia in the treatment of cancer, this neatly summarises results of relevant trials. The principles of the use of chemotherapy and the development of drugs are explained concisely, incorporating simple but effective diagrams.

Throughout this book, the text is well laid out with clear headings, making it easy to find information. The visual illustrations are, on the whole, easy to interpret and there are the obligatory selection of pictures of advanced malignancy to hold the readers' attention!

The chapter on bioimmunotherapy is packed with information; however, it is slightly overwhelming and may have benefited from diagrams to illustrate the salient points.

Chapters on tumour imaging and interventional radiology would be of particular interest to the nonradiologist. Understanding the uses and limitations of some of the techniques used in more depth should help the oncologist maintain a good relationship with their colleagues in radiology! The explanation of positron emission tomography (PET) scanning and its clinical uses is excellent.

The chapters on angiogenesis and gene therapy include enough detail to get a flavour of these subjects, plus provide references for further reading.

The second section of the book covers the management of the site-specific cancers, including the haematological malignancies, cancer in childhood and AIDS-related cancer. Although on closer inspection, there does not seem to be a chapter dedicated to the management of renal tumours.

Each chapter covers the pathology of the tumour with reference to the staging and classification systems in use. Clinical presentation and the different techniques used in diagnosis and staging are discussed. There is an up-to-date account of the treatment modalities that may be used in the treatment of each tumour type, whether that is surgery, chemotherapy or radiotherapy, or combinations of these. The explanations of chemotherapy regimens appear to be evidence based, with useful references to key trials and papers.

The planning and administration of radiotherapy is explained in good detail, with sensible use of visual illustrations. When read with reference to the chapters in the last section on radiotherapy planning techniques and conformal radiotherapy, this book stands as an ideal reference text for the trainee clinical oncologist.

The chapters include management of recurrent and metastatic disease, as well as some of the rarer tumours within each specific site.

Apart from comprehensive coverage of nonsurgical management of cancers, surgical principles of management are included. Of particular note are the general principles of surgery in head and neck cancer, and surgical approaches to treatment of liver tumours.

There is an interesting chapter on gestational trophoblastic tumours. The chapters on haematological malignancies, while perhaps not in-depth enough for the trainee haematologist, give a detailed insight for the oncologist or general physician.

Each chapter includes a short section on possible future directions for management and a highlighted box containing significant points that may be obtained from the chapter. There is also a box containing key references and then an extensive list of further references.

The third and final section is entitled management. This covers everything from symptom management to models of care, organisation of cancer services and cost analysis of nonsurgical cancer treatments.

There are chapters on symptom management and palliative care that give the reader a structured overview. In addition, there are separate chapters on communication issues and the holistic approach to cancer. These encourage a balanced approach to cancer management when slotted in between chapters on radiotherapy planning techniques.

There is an excellent chapter on brachytherapy that manages to make the physics of the subject understandable and clinically relevant!

The structure of clinical trials and the process of randomisation are explained in an easy to follow format. There is a chapter dedicated to medical audit, which summarises the requirements for a good audit.

In summary, this is a comprehensive text on the treatment of cancer. By its very nature, as new developments in treatment occur, some sections will become out of date. It is excellent as a standard text for the clinical oncologist, but also as a reference book for those working in other fields of medicine.

